# Supplementary material for: How to Form a Successful Team for the Novel Olympic Triathlon Discipline: The Mixed-Team-Relay
Source: J Funct Morphol Kinesiol. 2022 Jun 2;7(2):46. doi: 10.3390/jfmk7020046 (PMC9225130; doi:10.3390/jfmk7020046)
Supplement: Supplementary file 1 [file jfmk-07-00046-s001.zip › jfmk-1703581-supplementary.pdf]

Table S1. Sample

**Mean touch-zone value for each leg** in each ITU Triathlon Mixed Relay World Championship from 2014 to 2019						
	**2014**	**2015**	**2016**	**2017**	**2018**	**2019**
**Leg 1 (female)**	/	/	/	/	/	/
**Leg 2 (male)**	19 s	19 s	21 s	21 s	19 s	18 s
**Leg 3 (female)**	18 s	18 s	19 s	18 s	17 s	17 s
**Leg 4 (male)**	17 s	17 s	18 s	18 s	18 s	17 s
**Team**	54 s	54 s	58 s	57 s	54 s	52 s

**Number of finishing teams and athletes** in the ITU Triathlon Mixed Relay World Championships between 2014 and 2019			
**Year**	**Teams**	**Total Athletes**	**Athletes for gender**
**2014**	16	64	32
**2015**	16	64	32
**2016**	12	48	24
**2017**	18	72	36
**2018**	17	68	34
**2019**	13	52	26
**Total**	92	368	184

Table S2. Statistical Analysis Results

The results are showed following the order of Figures in the main text.

* Significantly different ($p \le 0.05$)
Pearson's r thresholds: .1 small (S), .3 moderate (M), .5 large (L), .7 very large (VL), .9 extremely large (EL) [9,10]

**Automatic linear regression: Leg times on relay time** (Figure 1 C)			
**Leg 1**	**Leg 2**	**Leg 3**	**Leg 4**
0.22	0.14	0.41	0.23
Accuracy: 99.6%			

**Automatic linear regression: Discipline/transition portion times on relay time** (Figure 2 A)						
	**Accuracy**	**Swim**	**T1**	**Cycle**	**T2**	**Run**
**Total**	97.9%	0.11	0.01	0.66	0.00	0.21
**G1**	95.0%	0.14	0.04	0.68	0.00	0.14
**Automatic linear regression: Discipline/Transition portion times on leg time** (Figure 2 B)						
	**Accuracy**	**Swim**	**T1**	**Cycle**	**T2**	**Run**
**Total**	99.8%	0.16	0.02	0.64	0.00	0.17
**G1**	100%	0.16	0.02	0.69	0.00	0.12

**Discipline percentages comparisons** (Figure 3 A-B)				
**Swim**	**T1**	**Cycle**	**T2**	**Run**
$H_{(3, 364)}= 5.899, p=0.117, \eta^2=.018$	$H_{(3, 364)}= 7.141, p=.068, \eta^2=.020$	$H_{(3, 364)}= 47.937, p=.000^*, \eta^2=.135^{S}$	$H_{(3, 364)}= 11.343, p=0.010^*, \eta^2=.029$	$H_{(3, 364)}= 58.150, p=0.000^*, \eta^2=.143^{S}$

	Cycle				***T2***				***Run***			
**Leg**	**1**	**2**	**3**	**4**	**1**	**2**	**3**	**4**	**1**	**2**	**3**	**4**
**1**		$U_{(182)}= 2145.000, p=.000^*, r=.432^{M}$	$U_{(182)}=2679.500, p=.000^*, r=.327^{M}$	$U_{(182)}=2008.000, p=.000^*, r=.459^{M}$		$U_{(182)}=3987.000, p=.497, r=.099$	$U_{(182)}=3942.500, p=.423, r=-.018$	$U_{(182)}=3286.500, p=.009^*, r=-.161^{S}$		$U_{(182)}=1964.000, p=.000^*, r=-.437^{M}$	$U_{(182)}=2858.000, p=.000^*, r=-.277^{S}$	$U_{(182)}=1898.000, p=.000^*, r=-.441^{M}$
**2**	$U_{(182)}= 2145.000, p=.000^*, r=.432^{M}$		$U_{(182)}=3712.000, p=.150; r=-.100^{S}$	$U_{(182)}=4044.500, p=.604; r=.037$	$U_{(182)}=3987.000, p=.497, r=.099$		$U_{(182)}=3809.000, p=.241; r=-.084$	$U_{(182)}=3110.000, p=.002^*; r=-.195^{S}$	$U_{(182)}=1964.000, p=.000^*; r=-.437^{M}$		$U_{(182)}=3154.000, p=.003^*; r=.239^{S}$	$U_{(182)}=4041.000, p=.597; r=-.027$
**3**	$U_{(182)}=2679.500, p=.000^*, r=.327^{M}$	$U_{(182)}=3712.000, p=.150; r=-.100$		$U_{(182)}=3554.000, p=.061; r=.134$	$U_{(182)}=3942.500, p=.423, r=-.018$	$U_{(182)}=3809.000, p=.241; r=-.084$		$U_{(182)}=3539.500, p=.055; r=-.137$	$U_{(182)}=2858.000, p=.000^*, r=-.277^{S}$	$U_{(182)}=3154.000, p=.003^*; r=.239^{S}$		$U_{(182)}=3013.000, p=.001^*; r=-.220^{S}$
**4**	$U_{(182)}=2008.000, p=.000^*, r=.459^{M}$	$U_{(182)}=4044.500, p=.604; r=.037$	$U_{(182)}=3554.000, p=.061; r=.134^{S}$		$U_{(182)}=3286.500, p=.009^*, r=-.161^{S}$	$U_{(182)}=3110.000, p=.002^*; r=-.195^{S}$	$U_{(182)}=3539.500, p=.055; r=-.137^{S}$		$U_{(182)}=1898.000, p=.000^*, r=-.441^{M}$	$U_{(182)}=4041.000, p=.597; r=-.027$	$U_{(182)}=3013.000, p=.001^*; r=-.220^{S}$	

**Discipline times comparisons: Same-sex** (Figure 3 C-F)					
	**Swim**	**T1**	**Cycle**	**T2**	**Run**
**Females**	$U_{(182)}= 4189.500, p=.906, r=-.033$	$U_{(182)}= 3772.000, p=.202, r=-.081$	$U_{(182)}= 2537.000, p=.000^*, r=.364^{M}$	$U_{(182)}= 4172.000, p=.866, r=.072$	$U_{(182)}= 3880.000, p=.330, r=-.043$
**Males**	$U_{(182)}= 2837.000, p=.000^*, r=.281^{S}$	$U_{(182)}= 4173.000, p=.870, r=-.011$	$U_{(182)}= 2623.000, p=.000^*, r=.317^{M}$	$U_{(182)}= 3806.000, p=.229, r=-.109^{S}$	$U_{(182)}= 2930.500, p=.000^*, r=.243^{S}$

**Leg times comparisons: Same-sex** (Figure 1 B)	
**Females**	**Males**
$U_{(182)}$=219.00, *p*=.000*, *r*=.400[M]	$U_{(182)}$= 3242.00, *p*=.006*, *r*=.255[S]

Leg comparisons: Percentage values on relay time (Figure 1 A)				
	**Leg 1**	**Leg 2**	**Leg 3**	**Leg 4**
**Leg 1**		$U_{(182)}$=.000, *p*=.000*, *r*=-.952[EL]	$U_{(182)}$=2461.500, *p*=.000*, *r*=.377[M]	$U_{(182)}$=1.000, *p*=.000*, *r*=-.919[EL]
**Leg 2**	$U_{(182)}$=.000, *p*=.000*, *r*=-.952[EL]		$U_{(182)}$=.000, *p*=.000*, *r*=.958[EL]	$U_{(182)}$=1226.000, *p*=.000*, *r*=.604[L]
**Leg 3**	$U_{(182)}$=2461.500*, *p*=.000, *r*=.377[M]	$U_{(182)}$=.000, *p*=.000*, *r*=.958[EL]		$U_{(182)}$=.000, *p*=.000*, *r*=-.934[EL]
**Leg 4**	$U_{(182)}$=1.000, *p*=.000*, *r*=-.919[EL]	$U_{(182)}$=1226.000, *p*=.000*, *r*=.600[L]	$U_{(182)}$=.000, *p*=.000*, *r*=-.934[EL]	
$H_{(3, 364)}$= 298.62, p=.000*, η^2=.894[VL]				
